# Exhaled Hydrogen after Lactulose Hydrogen Breath Test in Patient with Duodenal Ulcer Disease—A Pilot Study for *Helicobacter-pylori*-Associated Gastroduodenal Disease

**DOI:** 10.3390/life13010045

**Published:** 2022-12-23

**Authors:** Yi-Hsun Chen, Sharon Chia-Ju Chen, Jiunn-Wei Wang, Chiang-Shin Liu, Jeng-Yih Wu, Deng-Chyang Wu, Yu-Chung Su

**Affiliations:** 1 Division of Gastroenterology, Department of Internal Medicine, Kaohsiung Medical University Hospital, Kaohsiung Medical University, Kaohsiung City 80756, Taiwan; 2 Department of Medical Imaging and Radiological Sciences, Kaohsiung Medical University, Kaohsiung City 80756, Taiwan; 3 Department of Medicine, Faculty of Medicine, College of Medicine, Kaohsiung Medical University, Kaohsiung City 80756, Taiwan; 4 Department of Pathology, National Cheng Kung University Hospital, Tainan 70403, Taiwan

**Keywords:** *Helicobacter pylori*, lactulose hydrogen breath test, duodenal ulcer

## Abstract

Objectives: The precipitating mechanism(s) from the inactive to the active stage of duodenal ulcer disease (DU) is unclear. It has been shown that hydrogen gas from colonic fermentation provides an important energy source for *Helicobacter pylori* (Hp) colonization. The lactulose hydrogen breath test (LHBT) is a useful tool to assess the small intestinal and/or colon fermentation. This study examines the association(s) between the status of gastroduodenal disease and the result of a lactulose hydrogen breath test (LHBT). Materials and Methods: We enrolled Hp-positive active duodenal ulcer (aDU) patients, inactive DU (iDU) patients and patients with a positive Hp infection without structural gastroduodenal lesion, i.e., simple gastritis (SG Hp+). The patients with simple gastritis without Hp infection (SG Hp−) served as controls. Histological examinations of the gastric mucosa and lactulose hydrogen breath test (LHBT) were performed. Results: SG Hp+ patients tend to have advanced gastritis (pangastritis or corpus-predominant gastritis) compared with SG Hp− patients (7/29 vs. 0/14, *p* = 0.08). More iDU patients had advanced gastritis than either the SG Hp+ (7/9 vs. 7/29, *p* = 0.006) or aDU patients (7/9 vs. 6/24, *p* = 0.013). In comparison with the aDU patients, the iDU patients were also older (52.1 ± 12.6 vs. 42.2 ± 11.9 years, *p* = 0.02) and had a lower mean area under the curve value of the LHBT(AUC) (209.1 ± 86.0 vs. 421.9 ± 70.9, *p* = 0.023). Conclusion: aDU patients with a positive Hp infection have a lower grade of gastric mucosa damage than iDU patients and tend to have a higher level of exhaled hydrogen after LHBT.

## 1. Introduction

*Helicobacter pylori* (Hp) infection is known to be the causative agent for peptic ulcer disease, gastric-mucosa-associated lymphoid tissue lymphoma (MALToma), and the major factor for the development of atrophic gastritis in humans [1]. The pathophysiological mechanism of duodenal ulcers (DU) has been shown to derive from the loss of inhibitory control of acid secretion because of Hp-associated mucosa inflammation [2]. However, there is scarce information regarding the triggering factor(s) for recurrent duodenal ulcers in the presence of chronic Hp infection.

Hp-infected individuals have proved to be highly dominated by this organism concomitant with the relative depletion of other genera [3]. It remains unknown whether an alternation of microbiota composition can subsequently reshuffle the composition of the microbiome in the distant site. These changes could potentially result in detrimental changes in proinflammatory cytokines that lead to gastrointestinal mucosa damages [4]. On the other hand, previous observations in animals have reported that molecular hydrogen produced in the large intestine due to the fermentation reactions of sugar is a potential energy substrate for the sustention of Hp infection [5,6]. A recent investigation in animals also showed that the transport of the carcinogenic factor CagA into host cells is enhanced by the H2-utilizing respiratory chain of the bacterium [7]. These findings lead to the speculation that hydrogen produced from colon fermentation could be absorbed and delivered via vasculature to the distal mucosal site and serve as an enigma to bacterial survival.

Small intestinal bacterial overgrowth (SIBO) is the condition of an excessive number of symptom-causing bacteria in the small bowel, most of which are Gram-negative aerobic and anaerobic species that cause carbohydrates fermentation [8]. The oral intake of lactulose is normally not absorbed by the small intestine and fermented by bacteria to short-chain fatty acids as well as hydrogen and/or methane gas in the colon [9]. Clinically, lactulose hydrogen breath testing is often used to identify SIBO by the defined criteria. Although investigations have suggested that a substantial portion of abnormal breath test results implicate increasing colon fermentation instead of SIBO [10,11], both conditions may contribute to an increase in hydrogen production.

Applying the lactulose hydrogen breath test (LHBT) and histology examination, this study is designed to investigate the potential association of SIBO and/or the level of exhaled hydrogen among patients with Hp-positive active DU, inactive DU and simple gastritis (SG).

## 2. Materials and Methods

### 2.1. Patients, Inclusion, and Exclusion Criteria

Patients with dyspeptic symptoms and esophagogastroduodenoscopy (EGD) examinations were potential candidates for this study. The following patients were excluded: those with gastric ulcer(s), liver cirrhosis, a history of Hp eradication therapy, antibiotics, probiotics, proton pump inhibitor (PPI) or nonsteroid anti-inflammatory drug intake in the previous 3 months. Since Hp-negative duodenal ulcer disease involves heterogenous mechanisms [12], these patients were not included in the study. The enrolled patients were those with active ulcer(s) at the duodenal bulb (aDU), traction fold(s) and/or ulcer scar at the duodenal bulb in the absence of active ulcer (iDU), and those deprived of structural abnormalities of the gastroduodenal mucosa, i.e., simple gastritis (SG). This study was performed in line with the principles of the Declaration of Helsinki. All patients signed their informed consent. Approval was granted by the institutional review board of the Kaohsiung Medical University Hospital (KMUH-IRB-940242).

### 2.2. Esophagogastroduodenoscopy (EGD) Examination and Histology Grading

During the EGD examination, two sets of mucosal tissues were obtained from the gastric body and antrum. The specimens were fixed in 10% formalin, and a histological examination was performed to assess the severity of gastritis. The status of Hp infection was verified by the concurrent results of the rapid urease test by EGD examination and the subsequent urea breath test. Tissue damage was evaluated according to the topographic distribution of neutrophil activity [13] and was classified into four categories [14]: no gastritis (neutrophil activity: both antrum and body = 0); antrum-predominant gastritis (neutrophil activity: antral activity > body); pangastritis (neutrophil activity: antrum= body); and corpus-predominant gastritis (neutrophil activity: body > antrum). Advanced gastritis was defined as having histology findings of either pangastritis or corpus-predominant gastritis.

### 2.3. Lactulose Hydrogen Breath Test

The lactulose hydrogen breath test was performed on a separate day. After at least 12 h of fasting, the patients were instructed to drink 10 g of lactulose mixed with 100 cc of distilled water. The measurement of the baseline breath hydrogen levels and subsequent recording of the level of breath hydrogen were performed at 15 min intervals for 150 min. (QuinTron Instrument Company, Milwaukee, WI, USA). The presence of positive LHBT was defined according to the following criteria [15,16]:

1. An increase in hydrogen of ≥20 parts per million (p.p.m.) by 90 min;
2. Two peaks during the lactulose breath test with all peaks above 20 p.p.m.

For the purpose of a semiquantitative assessment, the AUC value of the exhaled hydrogen concentration in each test was also calculated from triangulated areas under the maximal increase in hydrogen concentration versus time from 0 to 150 min using the trapezoidal rule [17].

### 2.4. Statistical Analysis

Fisher’s exam and Mann–Whitney U test were used to compare the items between each category of the study subjects, including age, sex, Hp status, features of gastritis, and the results of the LHBT. The correlations between the AUC value of LHBT and other numerical data between each comparison were analyzed by logistic regression. A *p* value of < 0.05 was defined as significant. The statistical analysis was performed using IBM SPSS^®^ version 20.

## 3. Results

**Figure 1:**
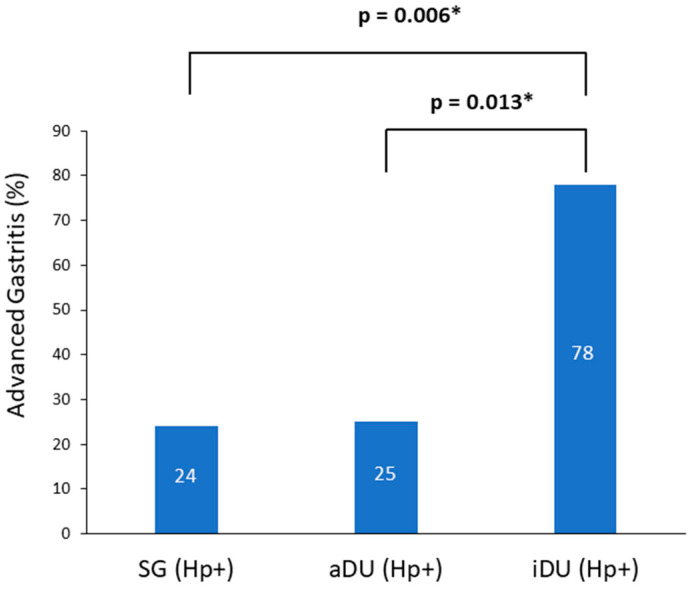
Prevalence of advanced gastritis among positive *Helicobacter pylori* patients. Notes: SG, simple gastritis; aDU, active duodenal ulcer; iDU, inactive duodenal ulcer; Hp, *Helicobacter pylori*. * *p* < 0.05.

**Figure 2:**
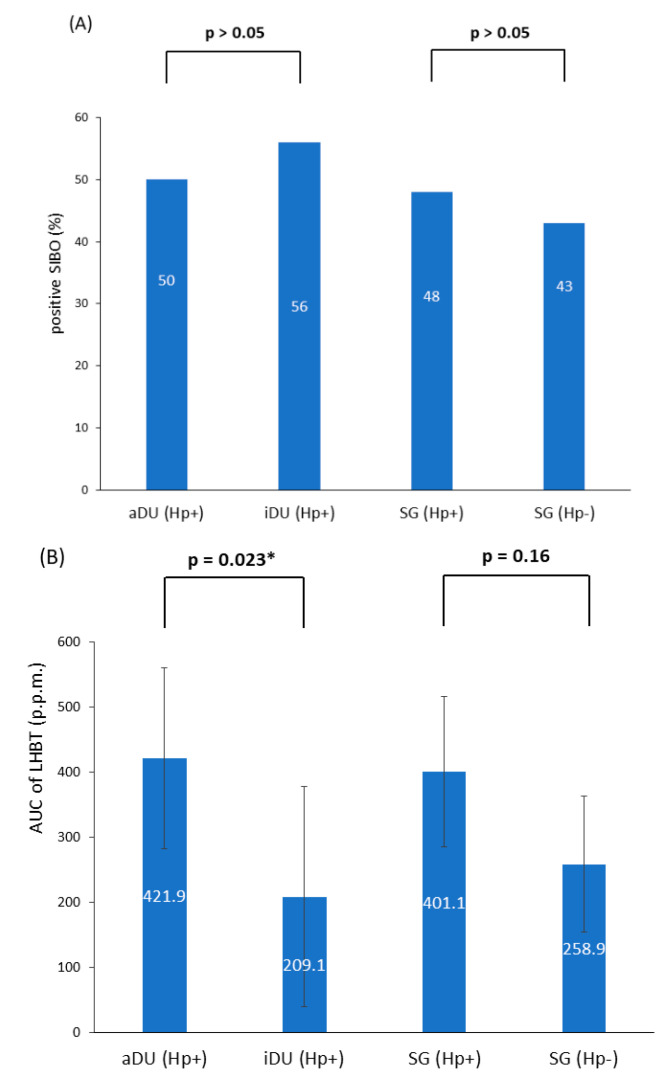
(**A**) The presence of SIBO within all subgroups (**B**) The mean AUC of LHBT within all subgroups. Notes: SIBO, small intestinal bacterial overgrowth; aDU, active duodenal ulcer; iDU, inactive duodenal ulcer; SG, simple gastritis; Hp, *Helicobacter pylori*; p.p.m., parts per million; LHBT, lactulose hydrogen breath test; AUC, area under the curve. * *p* < 0.05.

A total of 76 patients were recruited; of these, 39 patients were male and 37 were female, and their age ranged from 21 to 67 years. The demographic and clinical characteristics are shown in Table 1. The iDU patients appear to be significantly older and have more advanced gastritis than those of the aDU group (7/9 vs. 6/24, *p* = 0.013). The SG Hp− patients were devoid of either pangastritis or corpus predominate gastritis. The SG Hp+ patients showed a trend of advanced gastritis compared with the SG Hp– patients (7/29 vs. 0/14, *p* = 0.08). Figure 1 shows the prevalence rate and statistical significance of advanced gastritis among patients with a positive Hp infection. For the presence of SIBO and the mean AUC value of LHBT, the comparisons between each comparable group are shown in Figure 2. After adjusting the age, portion of advanced gastritis and AUC value, the features of gastritis independently distinguish between the iDU and aDU patients (Odds Ratio: 8.8, 95% Confidential Index: 1.1~70.0, *p* = 0.04).

The presence of SIBO was positively related with the AUC value of LHBT in patients with either positive Hp (aDU plus iDU plus SG Hp+: *n* = 62, mean AUC value, positive vs. negative SIBO: 490.8 vs. 271.8, *p* = 0.007) or negative Hp (SG Hp−: *n* = 14, mean AUC value, positive vs. negative SIBO: 411.3 vs. 144.7, *p* = 0.008), and each study cohort, respectively. In patients with Hp infection (*n* = 62), females had a higher mean AUC value of LHBT than males (486.5 vs. 276.0, *p* = 0.01). Within each disease group, the presence of SIBO or the mean AUC value of LHBT were not statistically significantly associated with the evaluated parameters such as gastritis or age.

## 4. Discussion

The present study assessed the potential association between the exhaled hydrogen after LHBT and the status of gastroduodenal disease in patients with Hp infection. The major finding was that the patients with aDU have a higher AUC value of exhaled hydrogen after LHBT than those with iDU, but this finding was confounded by the lower degree of gastric mucosa damage noted in aDU patients. Our findings are comparable with the results of the recent reports addressing the association between Hp infection and an altered colonic microbiota in animal [18] and human [19,20].

In this study, iDU patients have more advanced gastritis than those with either aDU or SG Hp+, and patients with SG Hp+ tend to have more advanced gastritis than SG Hp−. These results are consistent with previous reports that Hp infection is the major cause of antral gastritis [1,3,4]. The absence of a difference between DU and SG Hp+ patients in the feature of gastritis are comparable to a previous survey, showing the lack of histological distinction between patients with DU and SG [21]. However, in this study, there are significantly more iDU patients with advanced gastritis than SG Hp+. Strain-specific virulent factors have been linked to a more severe grade of mucosal inflammation that may promote the presence of duodenal ulcer disease [22]; however, studies from the East Asian strain have disclosed inconsistent results [23,24,25,26]. Conceivably, factors other than bacterial virulence [27], the adopted classification of gastritis or the activity of DU should be considered in the interpretation of these findings.

Hp-associated gastritis may potentially either increase or decrease acid secretion, which subsequently induces downstream effects on the small intestinal bacterial composition. Analogous to the effects of Hp-associated atrophic gastritis, the acid-suppressing effect of PPIs has been shown to increase the risk of SIBO [25] or alter the composition of both oropharyngeal and colonic microbiota species [28]. Our findings that aDU patients were less likely to have advanced gastritis are in line with the concept that aDU patients possess potent acid secretory despite of Hp infection [2]. In contrast, the iDU patients—who were noted to have more advanced gastritis that would be assumed to promote bacterial proliferation—had lower AUC value of exhaled hydrogen after LHBT compared with the aDU patients. Taking together the above findings suggests that either the lack of influence of Hp-associated gastropathy on the small intestine and/or colonic micromilieu, or other factors, could intervene. Hsu et al. reported that Hp eradication leads to a short-term increased relative abundance of Proteobacteria and decreased Firmicutes and Actinobacteria, but this returns to the pre-treatment level one year after treatment [29]. A multicenter follow-up study demonstrated that the eradication of Hp infection only minimally disrupted the microbiota one year after therapy [30]. In a recent study, Shah et al. demonstrated that PPI induced an increase in duodenal bacterial load without altering the level of exhaled hydrogen by a glucose hydrogen breath test [31].

Considering the findings that aDU patients have a higher semiquantitative level of exhaled hydrogen after LHBT than iDU patients, it could be argued that patients in the active stage of Hp-associated DU may have defective acid inhibition of gastric emptying [2], resulting in earlier colon fermentation of ingested lactulose that meets the defined criteria of SIBO [10,11]. However, there is no difference for the presence of SIBO between each comparable disease group in this study. SIBO signifies an increase in hydrogen production after lactulose challenge, whereas the AUC level of hydrogen represents the summation of both the baseline and increased level of exhaled hydrogen of LHBT. It should be noted that, in the present study, the presence of SIBO is related with a higher AUC value of LHBT in all the disease groups. A previous study showed that the optimal benefit of rifaximin was in subjects with irritable bowel syndrome (IBS) with abnormal baseline hydrogen levels during the lactulose breath test [17], implicating the important role of inherent gastrointestinal fermentation. If fermented hydrogen could be a potential energy substrate of Hp proliferation, a plausible proposition is that an increase in baseline plus challenged hydrogen production could augment antral inflammation in DU patients and result in a larger level of uninhibited acid secretion to provoke DU recurrence. The multivariable analysis in this study shows that a higher level of corpus gastritis independently distinguished the iDU patients from the aDU patients. This finding indicates that the occurrence of active duodenal ulcers depends more on the level of preserved gastric corpus mucosa that is reflective of acid secretory capacity. Therefore, in this study, a parallel increase in exhaled hydrogen level in the aDU patients—but not in iDU patients with more advanced gastritis—is more consistent with an upstream impact of intestinal fermentation on the Hp-associated gastropathy. A previous study demonstrated the association between the intensity of dyspeptic symptoms and the presence of Hp infection in IBS patients [32]. A meta-analysis disclosed that eradication therapy for Hp infection only improved symptoms in cohorts with concomitant IBS [33]. These findings warrant consideration of the interactions between the small intestine and/or colon microbiota and Hp infection.

In this study, the cause for the sex difference of increased exhaled hydrogen in DU patients is unknown and may require study with more cases to elucidate [34]. Given the decreasing prevalence of positive Hp aDU or iDU that may hamper the allocation of cases, the limitations of this pilot study are that we only incorporated small numbers of patients and employed a design of cross-sectional observation. Consequently, the power of the multivariable analysis and causative mechanism cannot be convincingly inferred. The current criteria of positive LHBT could be influenced by other factors, such as intestinal transit time [10,11] and colon dysbiosis [35]. Fermented gases other than hydrogen, such as methane, were not measured in this study [19,31,36].

## 5. Conclusions

In this pilot study, we found some association between the presence of altered exhaled hydrogen levels in patients with active duodenal ulcer disease. More studies will be required, including larger cohorts with longitudinal observations and newer techniques to measure colon fermentation [34] and evaluate the impact of gastric microbiota other than Hp [37].

## Figures and Tables

**Table 1:** Characteristics of the study cohorts.

	aDU Hp+ (*n* = 24)	iDU Hp+ (*n* = 9)	Statistics (*p*)	SG Hp+ (*n* = 29)	SG Hp− (*n* = 14)	Statistics (*p*)
Gender male/female (N)	14/10	5/4	1.0	12/17	8/6	0.52 ^a^
Age (years, Mean ± SD)	42.2 ± 11.9	52.1 ± 12.6	0.02 ^b^*	43.9 ± 9.3	39.4 ± 9.9	0.21 ^b^
Advanced gastritis (N)	6	7	0.013 ^a^*	7	0	0.08 ^a^
Positive SIBO (%)	50	56	0.71 ^a^	48	43	0.75 ^a^
AUC of LHBT (p.p.m., Mean ± SE)	421.9 ± 70.9	209.1 ± 86	0.023 ^b^*	401.1 ± 58.9	258.9 ± 53	0.16 ^b^

Notes: aDU, active duodenal ulcer; iDU, inactive duodenal ulcer; Hp, *Helicobacter pylori*; SG, simple gastritis; N, number; SD, standard deviation; SIBO, small intestinal bacterial overgrowth; LHBT, lactulose hydrogen breath test; AUC, area under the curve; SE, standard error. Note: ^a^ Fisher’s exact test; ^b^ Mann–Whitney U test, * *p* value < 0.05.

## Data Availability

Not applicable.
